# Integrated physiological and genomic analysis reveals structural variations and expression patterns of candidate genes for colored- and green-leaf poplar

**DOI:** 10.1038/s41598-019-47681-9

**Published:** 2019-08-01

**Authors:** Weibing Zhuang, Hongxue Wang, Tianyu Liu, Tao Wang, Fengjiao Zhang, Xiaochun Shu, Henghua Zhai, Zhong Wang

**Affiliations:** 1 Institute of Botany, Jiangsu Province and Chinese Academy of Sciences (Nanjing Botanical Garden Mem. Sun Yat-Sen), Nanjing, 210014 China; 2 College of Horticulture, Nanjing Agricultural University, Nanjing, 210095 China

**Keywords:** Plant sciences, Plant genetics, Plant molecular biology

## Abstract

Colored-leaf plants are increasingly popular and have been attracting more and more attentions. However, the molecular mechanism of leaf coloration in plants has not been fully understood. In this study, a colored-leaf cultivar of *Populus deltoides* (Caihong poplar, CHP) and green-leaf cultivar of *Populus deltoides* L2025 were used to explore the mechanism of leaf coloration through physiological and the whole genome resequencing analysis. The content of anthocyanins, total Chl, and carotenoids in the leaves of CHP and L2025 were evaluated. The ratio of anthocyanins to total Chl in CHP was 25.0 times higher than that in L2025; this could be attributed to the red leaf color of CHP. Based on the whole genome resequencing analysis, 951,421 polymorphic SNPs and 221,907 indels were screened between CHP and L2025. Using qRT-PCR analysis, three structural genes (flavonol synthase 1 family protein, UDP-glucose flavonoid 3-O-glucosyltransferase 3′ and flavonoid 3-O-galactosyl transferase family protein) and six transcription factors (MYB-related protein Myb4, transcription factor GAMYB, PtrMYB179, transcription factor bHLH53, transcription factor bHLH3, VARICOSE family protein) may be involved in the anthocyanin synthesis pathway, which could be used as candidate genes to explore the molecular regulation mechanism of leaf coloration in *Populus deltoids*, and could be used in molecular breeding in the future.

## Introduction

With the progress of society and economic development, colored-leaf plants are increasingly popular, which have a wide range of applications in the courtyard embellishment, road greening, garden set King and so on. The mechanism of leaf coloration in plants is quite complicated, and several factors such as the type, concentration, and distribution of pigments in leaf cells can affect the colors of leaves^1^. There are three kinds of pigments in the leaves of higher plants: (i) flavonoids, mainly including anthocyanins; (ii) carotenoids, mainly including lutein and carotenoid pigments; and (iii) chlorophyll (Chl), mainly including Chl a and Chl b^2^. In spring, the leaves of plants are very tender, and have a weak capacity for chlorophyll synthesis owing to the low temperature. Anthocyanins are often dominant in spring, making the leaves red^3,4^. The leaves of some woody trees turn red in fall as a result of the lower temperatures in this season; the lower temperatures are more conducive to the formation of anthocyanin pigments, and also mean the net concentration of Chl decreases as the leaves fall, increasing the ratio of anthocyanins to total Chl^5,6^. The anthocyanin content in purple-leaved Pak Choi and red-leaved Quanhong poplar (QHP) was much higher than the anthocyanin content in green-leaved Pak Choi and green-leaved cultivar of *Populus deltoides* L2025^7,8^. The occurrence of red, purple or blue leaves in many transgenic plants is due to the overexpression of the structural genes or regulatory genes associated with anthocyanin synthesis^9–12^.

The genes associated with the anthocyanin biosynthesis have been well studied; they include structural genes (*CHS*, *CHI*, *F3H*, *DFR*, *ANS*, and *UFGT*) and regulatory genes (MYB transcription factors, bHLH transcription factors, and WD40 transcription factors)^13^. These three kinds of transcription factors could combine to form a ternary complex to regulate structural genes or work individually^14–16^. The sequence changes in genes or their promoters may change the content of anthocyanins through the expression regulation of genes associated with anthocyanin synthesis. *MdMYB1–1* and *MdMYB1–2*, the alleles of *MdMYB1* associated with the coloration of apple fruit skin, has eight nucleotide differences at the promoter regions. These differences indicate that *MdMYB1–1* contributes to the red pigmentation in apple fruit skin, while *MdMYB1–2* does not contribute to the red pigmentation in apple skin^17^. An autoregulatory site produced due to the insertion of multiple repeats on the *MdMYB10* promoter, which produced increased anthocyanin content in flowers and fruits of apples through the increase in *MYB10* transcription levels^18^. In addition, plants overexpressing regulatory genes or structural genes associated with anthocyanins synthesis, especially the MYB family genes, could modulate expression of various genes involved in anthocyanin synthesis to modify the colors of plant tissues, such as flowers, leaves and fruits. Arabidopsis overexpressing *PAP1*/*AtMYB7*5 promoted the expression of structural genes associated with anthocyanin biosynthesis, leading to much higher accumulation of anthocyanins^19–21^. Tobacco overexpressing *IbMYB1a* promotes the concentration of anthocyanin through up-regulation of several structural genes, such as *ANS* and *DFR*^22^. Tobacco overexpressing *CsMYB6A* increased the expression levels of structural genes associated with flavonoid synthesis, such as *3GT* and *CHS*, leading to the high accumulation of anthocyanins in leaves of transgenic tobacco^11^. Recently, there have been larger number of reports on the functional analysis of genes associated with anthocyanin biosynthesis in poplar. Hybrid poplar overexpressing *PtrMYB119* and *PtrMYB118* separately, has higher anthocyanin content in leaves compared with those in wildtype poplar^23,24^. *MYB6*, is a R2R3-MYB transcription factor from *Populus tomentosa*, and transgenic MYB6-OE poplar showed a red color in the young leaves and shoots, indicating that overexpression of *MYB6* in transgenic poplar increased anthocyanin accumulation^25^. *Populus trichocarpa* R3 MYB-LIKE1 (*PtrRML1*), a repressor motif-containing poplar R3 MYB-like transcription factor, can reduce the anthocyanin content of stems, petioles, and rosette leaves of *PtrRML1* transgenic Arabidopsis plants^26^. Overexpression of *PtoMYB156*, *PtrMYB57*, *MYB182*, *MYB 165*, and *MYB 194* separately can also down-regulate anthocyanin biosynthesis in transgenic poplar^27–30^. Although genes associated with anthocyanin biosynthesis are well understood, the molecular mechanism of leaf coloration in poplar is not well understood, and the identification of candidate genes responsible for expression of leaf color in plants is a topic of research that is worth pursuing.

In the present study, colored-leaf cultivar of *Populus deltoides* (Caihong poplar, CHP) and green-leaf poplar L2025 were used to explore the mechanism of leaf coloration through physiological and whole genome resequencing analysis. The genes associated with anthocyanin biosynthesis and carrying more than five non-synonymous single nucleotide polymorphisms (SNPs) in the coding regions were identified as candidate genes. In addition, these candidate genes were further validated according to the quantitative reverse transcription polymerase chain reaction (qRT-PCR) analysis. These results could provide a physiological and global genomic perspective to understand the mechanism of leaf coloration in *Populus deltoides*, and could also contribute to the molecular breeding of poplar and other woody plants.

## Results

### Morphological phenotypes and growth status between CHP and L2025

**Figure 1:**
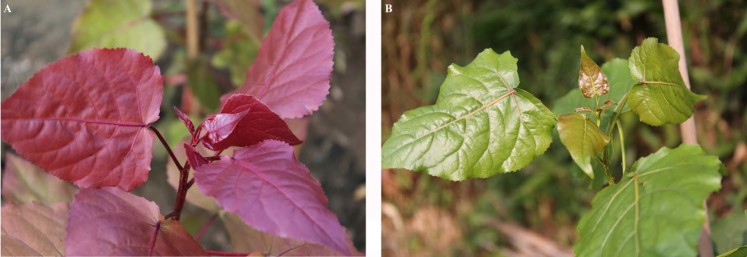
The morphological phenotype of CHP and L2025. (**A**) CHP; (**B**) L2025.

There are many significant differences in morphological phenotypes between CHP and L2025, especially with regarded to leaf color. The color of leaves, veins, branches and petioles in CHP are bright red, while the color of these in L2025 are always green (Fig. 1). For the propagated cuttings, CHP grew up to 172.3 cm height and 13.6 mm basal diameter in one year, while L2025 grew up to 387.5 cm height and 28.2 mm basal diameter (Table 1).

**Table 1:** The height and basal diameter of CHP and L2025, which were cutting propagation in one year.

	Height/cm ± SD	Basal diameter/mm ± SD
CHP	172.3 ± 3.0**	13.6 ± 0.2**
L2025	387.5 ± 2.3	28.2 ± 0.2

Each value is the mean ± SE of five replicates. ** and * indicate p < 0.001 and p < 0.05 by Student’s t-test, respectively.

### Changes of leaf pigment contents between CHP and L2025

There was a lower concentration of total Chl, Chl a, Chl b and carotenoids in CHP leaves compared with concentrations in L2025 leaves (Table 2). However, the concentration of anthocyanin in CHP leaves was much higher than concentrations in L2025 leaves (Table 2). The anthocyanin to total Chl content ratio in CHP leaves was 25.0 times of these in L2025 leaves; this may be the physiological reason why CHP had bright red leaves and weaker growth. The bright red leaves could be a consequence of the higher anthocyanin to total Chl ratio, and the weaker growth may be due to the lower total chlorophyll content.

**Table 2:** The content of chlorophyll, carotenoid and anthocyanin in the leaves of CHP and L2025.

	Chlorophyll a (mg/g FW)	Chlorophyll b (mg/g FW)	Total Chlorophyll (mg/g FW)	Carotenoids (mg/g FW)	Anthocyanin (mg/g FW)
CHP	0.60 ± 0.00**	0.29 ± 0.01**	0.89 ± 0.00**	0.14 ± 0.01**	386.51 ± 0.10**
L2025	1.02 ± 0.01	0.40 ± 0.01	1.42 ± 0.01	0.24 ± 0.00	24.63 ± 0.05

Each value is the mean ± SE of five replicates. ** and * indicate p < 0.001 and p < 0.05 by Student’s t-test, respectively.

### Whole genome resequencing of the *Populus deltoids* genome

To explore the genome variation between CHP and L2025, the whole genome resequencing was conducted using the *P*. *trichocarpa* genome as a reference. A total of 106.4 million reads (about 15.96 Gb) for L2025 and 108.9 million reads (16.33 Gb) for CHP were generated compared with the reference genome, which has 37.2× and 38.4× sequencing depth, respectively (Table 3). There was 410.4 Mb consensus sequence in the L2025 genome from 100.7 million reads (94.7% of the total reads), and 410.8 Mb consensus sequence in the CHP genome from 103.5 million reads (95.0% of the total reads). The CHP and L2025 genome coverage were 95.8% and 95.7%, respectively, compared with the *P*. *trichocarpa* genome (Table 3). Among 36,071 homozygote polymorphic SNPs, transitions (C/T and A/G) accounted for 65.7%, and transversions (A/T, A/C, G/T, and C/G) accounted for 34.3%, indicating that transitions occurred more easily than transversions (Table S1).

**Table 3:** The screening of genome variation between CHP and L2025 compared with the reference genome based on the whole genome resequencing.

	L2025	CHP
Total reads	106,415,483	108,920,101
Total size (bp)	4,723,926,206	4,876,051,559
Sequencing depth	37.2371x	38.4297x
Mapped reads	100,732,805 (94.7%)	103,464,008 (95.0%)
Properly paired reads	98,599,707	101,402,196
Consensus sequence (bp)	410444509	410845308
Genome coverage	95.7%	95.8%

**Figure 2:**
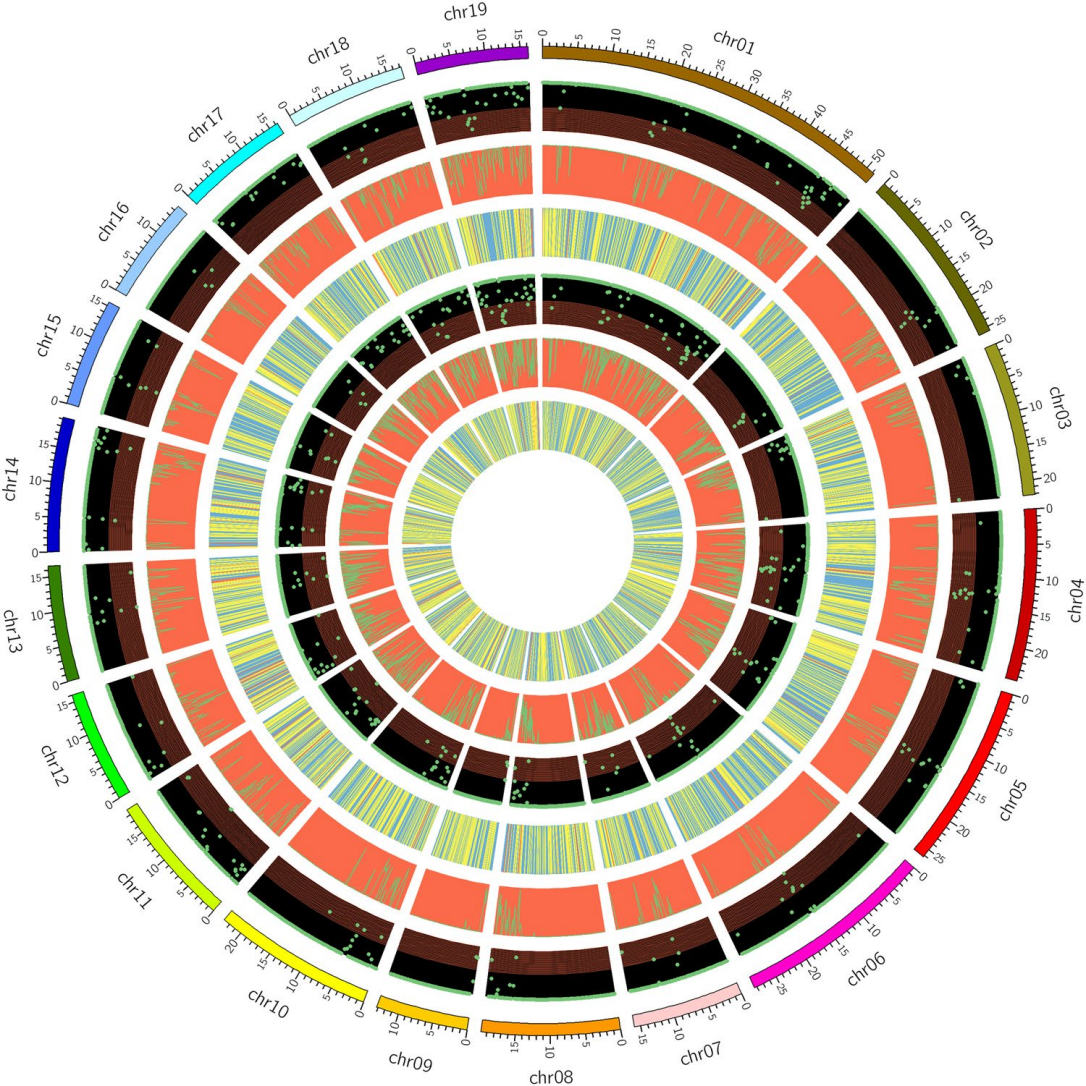
The distribution of SNPs, indels and Structural variation (SV) in each poplar chromosome of CHP and L2025 compared with the chromosome of *Populus trichocarpa*. Structural variation (SV) in genome includes insertion, duplication, deletion, translocation and inversion with length of more than 50 bp. From outside to inside in turn is chromosome location, distribution of SNP density, indels density and SV in each poplar chromosome of L2025 and CHP, respectively.

There were 951,421 polymorphic SNPs between L2025 and CHP compared with the *P*. *trichocarpa* genome. The highest number of SNPs in chromosome 01 was 94,154, and the lowest number of SNPs in chromosome 16 was 32,315 (Fig. 2, Table S2). Among these polymorphic SNPs, 33.2% of total polymorphic SNPs occurred in genic regions, and the remaining ones occurred in intergenic regions. There were 91,732 polymorphic SNPs in coding sequence regions, and non-synonymous and synonymous SNPs accounted for 53.4% and 46.6%, respectively. The functions of 36,389 genes might be changed due to the polymorphic SNPs occurred in coding sequence regions (Table S2).

There was a similar trend for the frequency and length of deletions and insertions. The most frequent indels were mononucleotide indels, accounting for 49.9% of the total. The next most frequent were dinucleotide indels with 18.1%, followed by trinucleotide indels with 9.7%, and tetranucleotide indels with 7.2% (Fig. S1). Among the 221,907 polymorphic indels, the highest number of polymorphic indels was 21,607 on chromosome 01, and the smallest number of polymorphic indels was 7580 on chromosome 16 (Table S3). Most of the polymorphic indels (71.2%) occurred in intergenic regions. Of the 63,832 polymorphic indels in genic regions, there were 45,596 indels in the intron regions, 13,511 indels in the untranslated regions, and 4725 indels in the coding sequence regions (Table S3).

### Gene ontology and KEGG analysis of candidate genes with polymorphic SNPs

**Figure 3:**
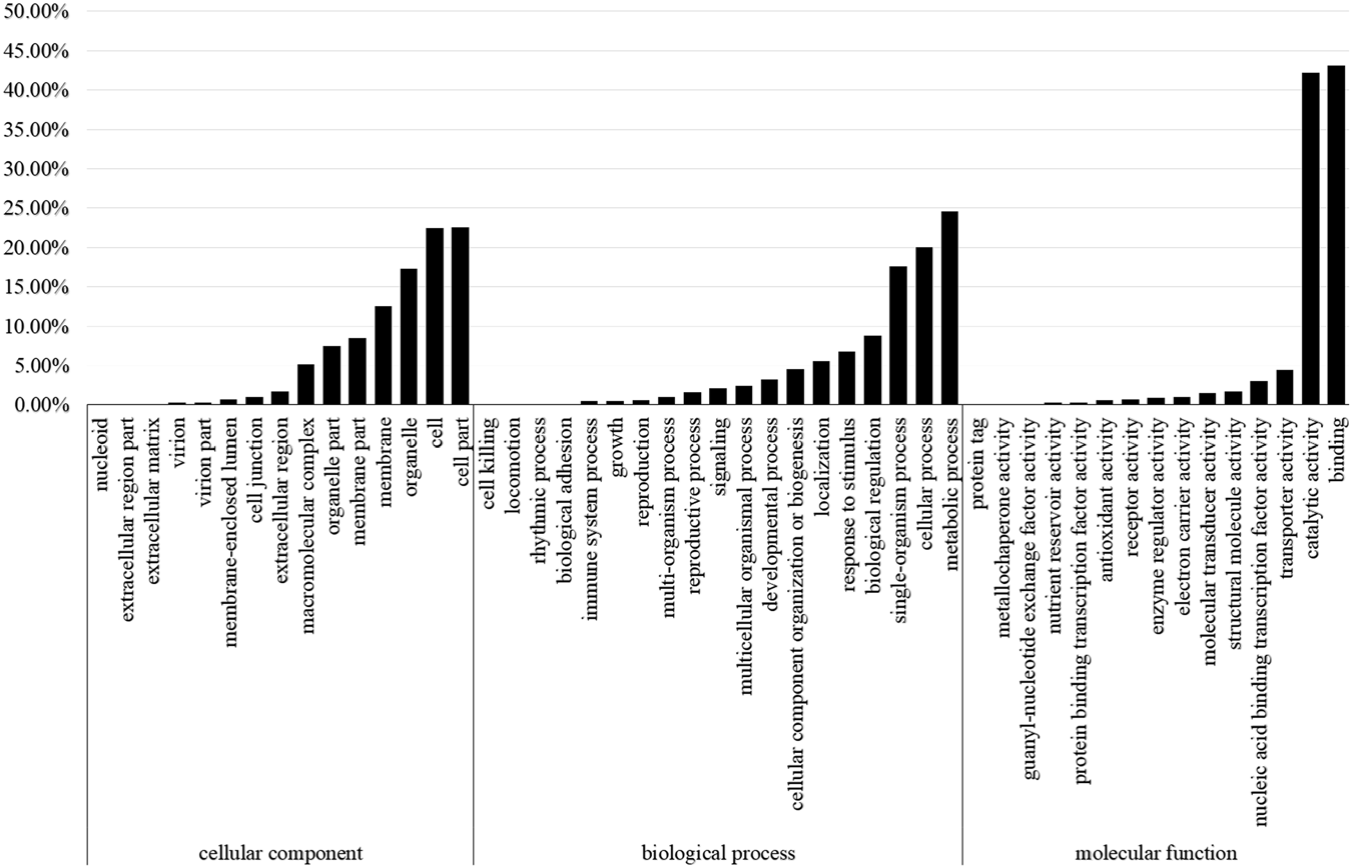
Gene ontology (GO) functional enrichment analysis of candidate genes carrying polymorphic SNPs between CHP and L2025.

Gene Ontology (GO) analysis of candidate genes with polymorphic SNPs was conducted between L2025 and CHP. For the ‘molecular function’, the binding contained most genes (13,856), followed by catalytic activity (13,577) and transporter activity (1444). There were 968 genes for nucleic acid binding transcription factor activity, 551 genes for structural molecule activity, and 477 genes for molecular transducer activity. These candidate genes were also classified according to the ‘cellular components’ as cell part with 10,445 genes, membrane with 5797 genes, organelle with 7992 genes, and cell with 10,409 genes. For the ‘biological processes’, the metabolic processes contained 16,657 genes, cellular processes contained 13,573 genes, and single-organism processes contained 11,918 genes (Fig. 3).

**Figure 4:**
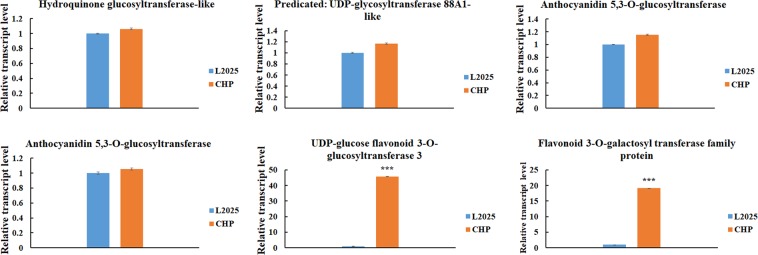
The relative expression levels of six genes involved in flavonoid biosynthesis in the leaves determined by quantitative real-time PCR (qRT-PCR) analysis between L2025 and CHP. Bars indicated the mean ± SE, n = 3. ***Indicated P ≤ 0.001.

The KEGG pathway enrichment analysis of candidate genes were conducted, and ‘Flavonoid biosynthesis’ (ko00941) was the enriched pathway associated with anthocyanin biosynthesis. There were 27 genes carrying polymorphic SNPs in this pathway. To further screen the possibly useful candidate genes associated with anthocyanin biosynthesis, genes carrying more than five non-synonymous SNPs in coding regions were considered. If these candidate genes do not work, genes carrying less than five non-synonymous SNPs in coding regions were also considered. In the flavonoid biosynthesis pathway, flavonol synthase 1 family protein (*Ptr FLS1*, Potri.004G139500) and shikimate O-hydroxycinnamoyl transferase-like (Potri.005G028000), carried more than five non-synonymous SNPs in coding regions, and were considered as candidate genes (Table 4). The relative expression level of these two candidate genes was evaluated, and there was a significant difference for *Ptr FLS1* between CHP and L2025, and no significant difference for Shikimate O-hydroxycinnamoyl transferase-like (Fig. S2). Therefore, *Ptr FLS1* was screened as a candidate gene associated with anthocyanin biosynthesis. In addition, UDP-glucosyl transferase family genes with polymorphic SNPs were also counted. Six genes with more than five non-synonymous SNPs in coding regions are shown in Table S4. The relative expression level of these six genes was also evaluated by qRT-PCR, and there were significant differences between UDP-glucose flavonoid 3-O-glucosyltransferase 3 and flavonoid 3-O-galactosyl transferase family protein; there were no significant differences among the other four genes (Fig. 4). Therefore, UDP-glucose flavonoid 3-O-glucosyltransferase 3 and flavonoid 3-O-galactosyl transferase family protein were also considered as candidate genes associated with anthocyanin biosynthesis.

**Table 4:** The statistics of genes involved in the flavonoid biosynthesis carrying polymorphic SNPs in coding regions between CHP and L2025.

Gene ID	SNP numbers in coding regions	Description
Potri.013G073300	0	trans-cinnamate 4-hydroxylase [*Populus tremuloides*]
Potri.018G146100	0	trans-cinnamate 4-hydroxylase [*Populus trichocarpa*]
Potri.014G145100	0	chalcone synthase [*Populus alba*]
Potri.010G104400	0	caffeoyl-CoA O-methyltransferase-like [*Populus euphratica*]
Potri.009G069100	0	flavonoid 3′,5′-hydroxylase 2 isoform 1 [*Theobroma cacao*]
Potri.005G229500	0	dihydroflavonol-4-reductase [*Vitis vinifera*]
Potri.004G139700	0	flavonol synthase 1 family protein [*Populus trichocarpa*]
Potri.003G176700	0	chalcone synthase 1-like isoform X1 [*Populus euphratica*]
Potri.003G119100	0	leucoanthocyanidin dioxygenase-like [*Populus euphratica*]
Potri.002G127500	0	dihydroflavonol-4-reductase [*Zea mays*]
Potri.002G033600	0	dihydroflavonol reductase [*Populus tremuloides*]
Potri.001G274600	0	flavonoid 3′,5′-hydroxylase 2 [*Petunia hybrida*]
Potri.015G050200	1	leucoanthocyanidin reductase [*Desmodium uncinatum*]
Potri.012G138800	1	stilbene synthase 4 OS = (Grape) PE = 3 SV = 1 [*Vitis vinifera*]
Potri.003G176800	1	naregenin-chalcone synthase family protein [*Populus trichocarpa*]
Potri.001G051600	1	naregenin-chalcone synthase family protein [*Populus trichocarpa*]
Potri.001G051500	1	naregenin-chalcone synthase family protein [*Populus trichocarpa*]
Potri.013G073300	1	flavonoid 3′-monooxygenase [*Petunia hybrida*]
Potri.005G113700	2	naringenin-2-oxoglutarate 3-dioxygenase [*Vitis vinifera*]
Potri.005G113900	2	naringenin-2-oxoglutarate 3-dioxygenase [*Vitis vinifera*]
Potri.010G129800	2	leucoanthocyanidin reductase [*Desmodium uncinatum*]
Potri.010G213000	2	chalcone isomerase family protein [*Populus trichocarpa*]
Potri.003G176900	3	PREDICTED: chalcone synthase 1 [*Populus euphratica*]
Potri.003G183900	3	shikimate O-hydroxycinnamoyltransferase [Arabidopsis thaliana]
Potri.004G030700	3	leucoanthocyanidin reductase family protein [*Populus trichocarpa*]
Potri.005G028000	5	shikimate O-hydroxycinnamoyltransferase-like [*Populus euphratica*]
Potri.004G139500	9	flavonol synthase 1 family protein [*Populus trichocarpa*]

### Candidate transcription factors associated with anthocyanin biosynthesis between L2025 and CHP

**Figure 5:**
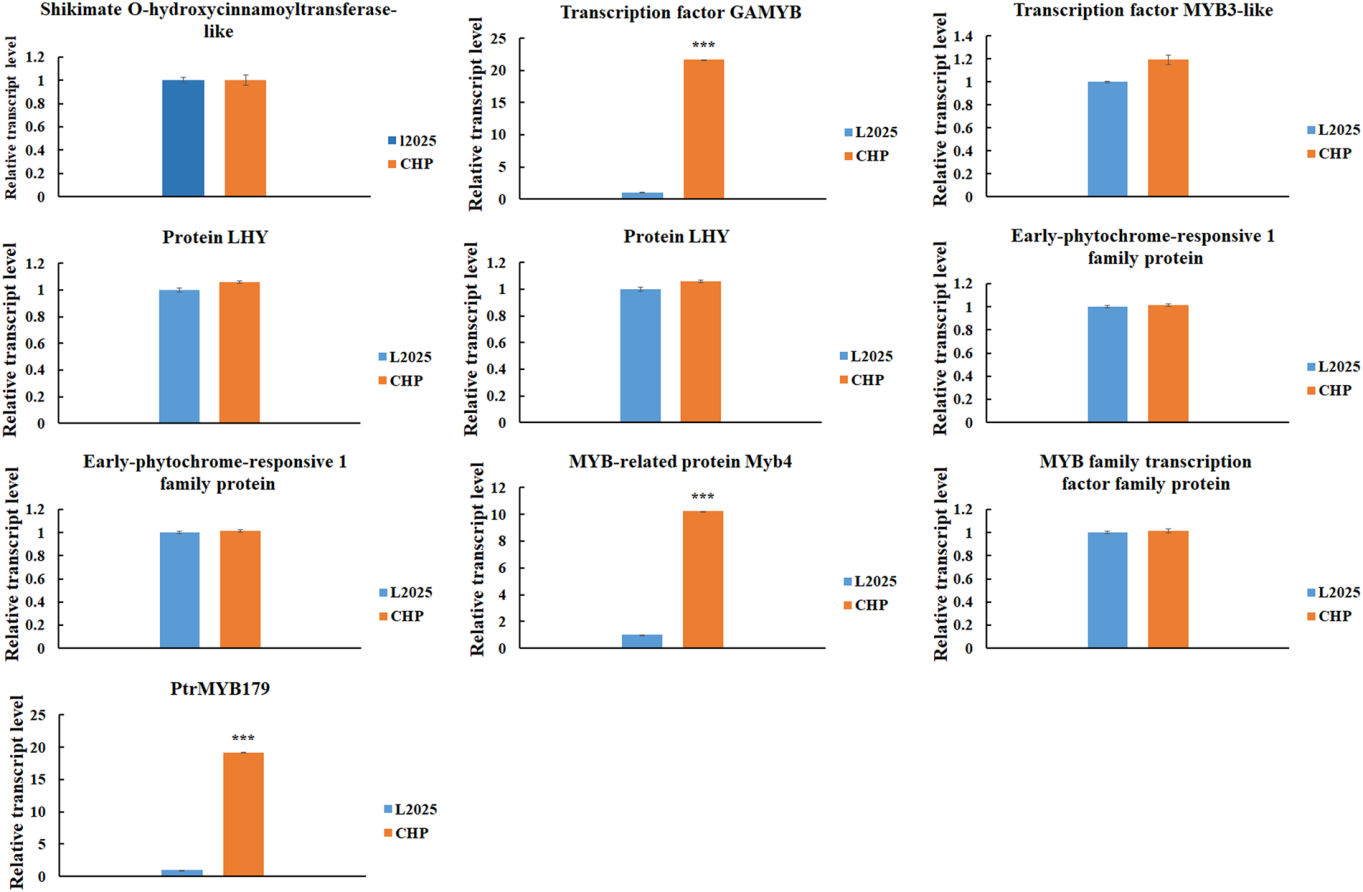
The relative expression levels of ten R2R3-MYB family genes in the leaves determined by quantitative real-time PCR (qRT-PCR) analysis between L2025 and CHP. Bars indicate the mean ± SE, n = 3. ***Indicated P ≤ 0.001.

**Figure 6:**
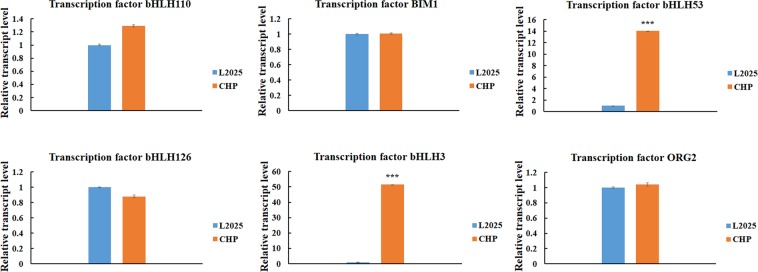
The relative expression levels of six bHLH family genes in the leaves determined by quantitative real-time PCR (qRT-PCR) analysis between L2025 and CHP. Bars indicate the mean ± SE, n = 3. ***Indicated P ≤ 0.001.

**Figure 7:**
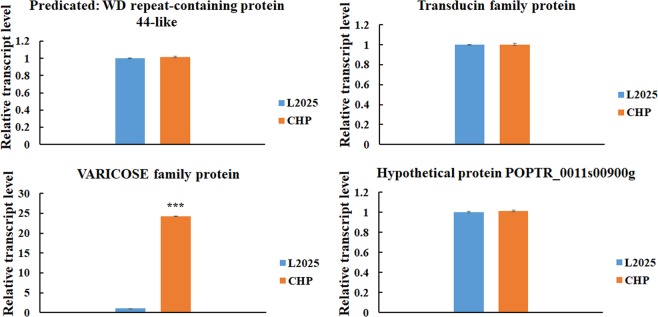
The relative expression levels of four WD40 family genes in the leaves determined by quantitative real-time PCR (qRT-PCR) analysis between L2025 and CHP. Bars indicate the mean ± SE, n = 3. ***Indicated P ≤ 0.001.

The anthocyanin biosynthesis was mainly affected by three families of transcription factors: MYB, bHLH, and WD40. There were 157 MYB family genes, 121 bHLH family genes, and 57 WD40 family genes. Ten candidate transcription factors with more than five non-synonymous SNPs in coding regions were identified for MYB family genes, six for bHLH family genes, and four for WD40 family genes (Tables S5–S7). To further screen candidate transcription factors associated with anthocyanin biosynthesis, the relative expression levels of these ten, six, and four candidate transcription factors were conducted. There was much significant difference for ‘MYB-related protein Myb4’, ‘PtrMYB179’ and ‘transcription factor transcription factor GAMYB’ of leaves between L2025 and CHP, and no significant difference for the left ones (Fig. 5). There was higher relative expression of ‘transcription factor bHLH53’ and ‘transcription factor bHLH3’ in CHP leaves compared with that in L2025 leaves, and no significant differences for the other transcription factors (Fig. 6). For the WD40 family genes, there were significant differences for expression of the ‘VARICOSE family protein’ in CHP leaves compared with L2025 leaves; there were no significant differences for the other transcription factors (Fig. 7). These three MYB family, two bHLH family, and one WD40 family genes might play important roles in determining coloration of poplar, and could provide some references to further explore the molecular mechanisms of anthocyanin biosynthesis in poplar and other woody plants.

### Relative expression levels of genes associated with anthocyanin biosynthesis in CHP

**Figure 8:**
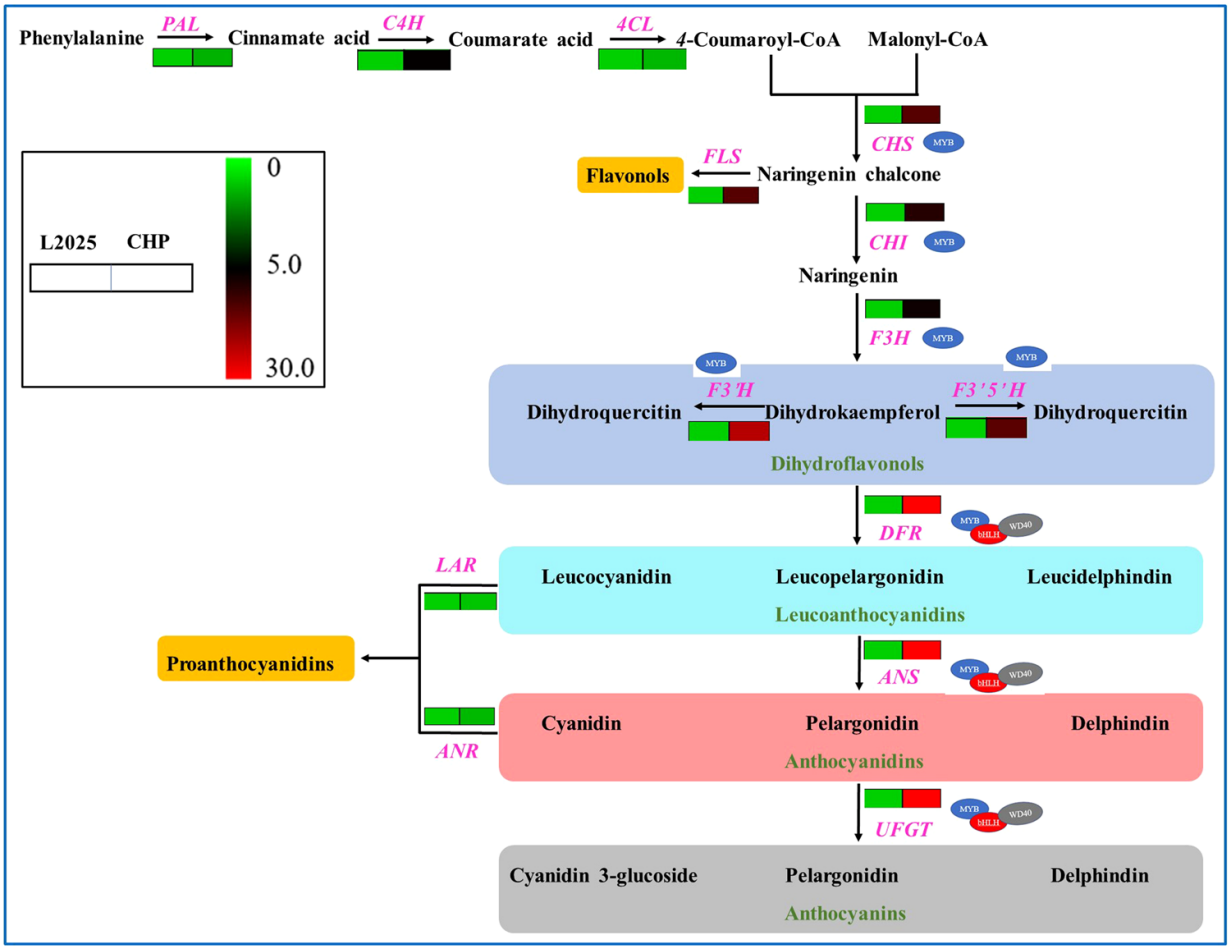
The relative expression levels of genes associated with anthocyanin biosynthesis in the leaves of CHP and L2025. The coloration of scale indicates the relative expression levels of genes. Red color indicates high relative expression levels of genes, and blue indicates low relative expression levels of genes. The relative expression levels of gene on left and right are from the leaves of CHP and L2025.

There were three structural genes associate with anthocyanin biosynthesis in our results, which had more than five non-synonymous SNPs in coding regions and significant differences in the relative expression levels between CHP leaves and L2025 leaves. To better discover the regulation mechanism of anthocyanin biosynthesis, the relative expression levels of other structural genes were also evaluated. Our findings showed higher relative expression levels in CHP leaves for the structural genes compared with L2025 leaves (Fig. 8). Compared with the relative expression difference of anthocyanin biosynthesis upstream genes such as *4CL*, *CHS*, and *F3H* between CHP and L2025, there were much greater differences in the relative expression difference of anthocyanin biosynthesis downstream genes such as *UFGT*, *DFR*, and *ANS*, which could be attributed to the higher content of anthocyanins in CHP leaves.

## Discussion

The different colors that appear in various plant tissues are determined by the ratio and distribution of three kinds of pigments (chlorophyll, carotenoids, and flavonoids/anthocyanins). In green-leaved plants, there is a high ratio of chlorophyll to the other two pigments; in yellow-leaved plants, there is a high ratio of carotenoids to the other two pigments; and in red-, purple-, and blue-leaved plants there is a high ratio of anthocyanins to the other two pigments^1^. In the present study, a higher ratio of anthocyanins to the other two pigments was observed in the red leaves of CHP, compared with in the green leaves of L2025 (Table 2). In a previous study, a high ratio of anthocyanins to the other two pigments was observed in the purple leaves of the Quanhong *P*. *deltoides* cultivar (QHP)^8^. The red or purple leaf color of many transgenic plants has been associated with the overexpression of genes associated with anthocyanin synthesis^11,22,23^. Therefore, the high ratio of anthocyanins content to the other two pigments in CHP may be the physiological reason for the bright red leaves of CHP.

In addition, the growth of CHP was inhibited compared with the growth of L2025 (Table 1); in previous studies, the growth of purple-leaved QHP was also less than that of L2025^8^. The transgenic tomato plants overexpressing *AtPAP2* accumulated much more anthocyanins compared with the wildtype, and were also significantly smaller^31^. Poplars overexpressing *PtrMYB119* accumulated more anthocyanins compared with the wildtype; however, growth of transgenic poplars was similar with the wildtype^23^.

### The structural genes involved in anthocyanin biosynthesis of leaves in CHP

The genes involved in anthocyanins biosynthesis including early biosynthetic genes (*CHS*, *CHI*, *F3H*, *F3*′*H*, and *F3*′*5*′*H*) and late biosynthetic genes (*DFR*, *ANS*, and *UFGT*) have been well studied^13^. In the present study, many genes carrying polymorphic SNPs were found to be involved in the flavonoid biosynthesis pathway, such as naregenin-chalcone synthase family protein, chalcone synthase 1, chalcone isomerase family protein, leucoanthocyanidin reductase family protein, dihydroflavonol reductase, leucoanthocyanidin dioxygenase-like (Tables 4 and S4). There were eight genes with more than five non-synonymous SNPs in the coding regions. Three genes (flavonol synthase 1 family protein, UDP-glucose flavonoid 3-O-glucosyltransferase 3, and flavonoid 3-O-galactosyl transferase family protein) had much greater differences in the relative expression levels between CHP and L2025 leaves and could be considered as candidate genes associated with anthocyanin biosynthesis. There were nine, non-synonymous SNPs in coding regions of flavonol synthase 1 family protein, five in coding regions of UDP-glucose flavonoid 3-O-glucosyltransferase 3, and fifteen in coding regions of flavonoid 3-O-galactosyl transferase family protein (Tables 4 and S4). Such mutations always induced abnormal anthocyanin accumulation, and led to the colored plants. The mutation of *ANS* or *DFR* genes led to the formation of pink onions^32,33^ and beans with a black seed coat was produced owing to the deletion of the *CHS* promoter^34,35^. The deletion of *DFR* can also lead to purple ornamental kale^36^. As flavonol synthase 1 family protein, UDP-glucose flavonoid 3-O-glucosyltransferase 3, and flavonoid 3-O-galactosyl transferase family protein have more than five non-synonymous SNPs in the coding regions and significant differences in relative expression levels, these three genes can be used as candidate genes to further explore the molecular mechanism of leaf coloration in poplar and other colored-leaf plants.

### The transcription factors involved in anthocyanin biosynthesis of leaves in CHP

Three kinds of transcription factors, R2R3-MYB, bHLH, and WD40 transcription factors, are crucial for anthocyanin accumulation; R2R3-MYB transcription factors seem to be especially important^37^. *PAP1* and *PAP2* in Arabidopsis, belonging to R2R3-MYB transcription factors, can enhance anthocyanin production by up-regulation of structural genes such as *LDOX*, *DFR* and *CHS*^19,37^. Arabidopsis overexpressing *PAP1*/*AtMYB75* promoted anthocyanin biosynthesis by enhancing the expression levels of structural genes^20^. UDP-Glc: flavonoid 3-O-glucosyltransferase (*UFGT*) gene in grape was induced through the expression of *VvMYBA1* and *VvMYBA2*, and the anthocyanin biosynthesis capability was also enhanced^38–40^. Tobacco overexpressing *VvMYB5b* accumulated much more anthocyanidin and proanthocyanidin in flowers by increasing the expression levels of structural genes, such as *F3H*, *CHI*, and *ANS*^41^. A dual-pigmented sweet potato with higher content of carotenoids and anthocyanins was generated owing to the over-expression of *IbMYB1a* in a single orange-fleshed sweet potato cultivar^42^. The almost capabilities variation of anthocyanin biosynthesis in *Citrus* species were the differences of MYB transcription factor activity^43^. *MdbHLH3* and *MdWD40* can bind *MdMYB10* to increase its activity, which further enhance the anthocyanin biosynthesis^44,45^. *MdbHLH3* can also interact with the promoter of *MdMYB9* and *MdMYB11* to promote its transcription activity^22^. bHLH and WD40 can combine with MYB to form MYB–bHLH–WD40 ternary complexes to better regulate anthocyanin biosynthesis^46,47^. The MYB involved in anthocyanin regulation in Arabidopsis has been well studied, and *PAP1*, *PAP2*, *AtMYB113*, and *AtMYB114* play critical roles during the anthocyanin synthesis. The phylogenetic analysis indicated that PtrMYB179 was clustered into the same group with *PAP1* and *PAP2* (Fig. S3). In addition, *PtrMYB179* in the present study is the same with *PtrMYB117* in the study conducted by Cho, and over-expression of *PtrMYB119* was reported to enhance anthocyanin accumulation and produce the colored-leaf poplar^23^. Therefore, ‘MYB-related protein Myb4’, ‘PtrMYB179’ and ‘transcription factor GAMYB’ might play critical roles in the synthesis of anthocyanin in CHP.

Except for the effects of expression levels of MYB family genes on anthocyanin synthesis, the mutations of MYB family genes also lead to the abnormal anthocyanin synthesis. In wheat, there is a 19 bp deletion in the coding sequence of *TaMYB10-B*, which contributed to the white grain^48^. In wild strawberry, one single nucleotide mutation in *FvMYB10* was reported to cause yellow fruit^49^. *MrMYB1* is involved in anthocyanin accumulation in the Chinese bayberry fruit, and a nonsense mutation in the *MYB1* protein is responsible for no or low expression of *MYB1* in the white and red fruit^50^. The deletion of two exons of the gene coding for *DFR* determined the color difference between yellow and red onions, and active red and inactive yellow *DFR* alleles have been designated as *DFR-AR* and *DFR-ATRN*, respectively^51^. There was a high level of the Riant protein in red flowers of peach, and barely detectable levels of the Riant protein in variegated flowers; this was attributed to small insertions and deletions (indels) in the last exon, leading to a frameshift mutation^2^. In the present study, the expression level of three MYB family genes, two bHLH family genes, and one WD40 family gene was much higher in CHP leaves than in L2025 leaves.

The present study investigated the physiological mechanism of leaf coloration in poplar. The candidate genes associated with anthocyanin biosynthesis were also identified. The genes can be used as target genes to acquire the desired color poplar through the Crispr/Cas 9 system, RNA interference or overexpressing system, and obtain more genes which can change the color of plant leaves. Taken together, our findings provide important information for the designing of molecular markers associated with color traits, and have considerable potential to assist in the breeding of colored-leaf poplar and other woody plants in the future.

## Materials and Methods

### Plant materials

Two *Populus deltoids* cultivars, CHP with bright red leaf and wild-type L2025 with green leaf were cultivated at the experimental field of Nanjing botanical garden Mem. Sun Yat-Sen (32°3′N, 118°49′E) in April 2017. Both CHP and L2025 were cutting propagation. To explore the mechanism of leaf coloration in poplar, the leaves were collected from CHP and L2025 trees on June 5, 2017 to determine the physiological index, such as the content of anthocyanins and chlorophyll, and the changes of molecular aspects, such as genomic polymorphic SNPs and the expression levels of candidate genes. Five seedlings of CHP and L2025 were selected, and five leaves per plant were collected. The leaves were stored at −80 °C after putting them into liquid nitrogen for fast frozen to conduct the subsequent analysis.

### The difference of growth status between CHP and L2025

The heights and stem diameters (about 100 cm above the soil) were determined after their defoliation (15 December, 2017). Duncan’s multiple range test were conducted for the statistical analyses in SPSS 17.0 (SPSS Inc., Chicago, IL).

### The determination of chlorophyll, carotenoid and anthocyanin in the leaves of CHP and L2025

To determine the chlorophyll content of leaves in CHP and L2025, about 0.1 g leaves was grinded, and the powers were put into 95% ethanol (5 mL) for 2 h at 50 °C. The mixture was centrifuged at 5,000 g for 5 min, after that the supernatant was obtained, which was used to measure the absorbance with a spectrophotometer at 470, 649, and 665 nm. The chlorophyll content was computed according to the method described by Lichtenthaler and Wellburn^52^. To determine the total anthocyanin content of leaves in CHP and L2025, about 1.0 g of the fresh leaves was immersed into 1% (v/v) HCl ethanol (10 mL) at 60 °C for 30 min. The mixture was centrifuged at 13,000 g for 5 min, after that the supernatant was obtained, which was used to measure the absorbance with a spectrophotometer at 530, 620 and 650 nm. The content of total anthocyanin was measured based on the method described by Yue^53^.

### Construction of DNA library and massively parallel sequencing

Genomic DNA was extracted from the leaves of CHP and L2025 with the modified method from Kim^54^. After that, the whole genome resequencing was conducted by Genepioneer Biotechnologies (Nanjing, China) on an Illumina HiSeq4000™. The standard Illumina protocol was used to construct DNA library and subsequent sequencing. Paired-end sequencing libraries was purified using the QIA quick PCR kit, which has an insertion of about 200 to 400 bp. The clusters were generated after end repair with poly-A on the 3´ ends and the ligated adaptors. The fragments were selected based on the agarose gel electrophoresis, and amplified by the PCR. The fragments with 2 × 150 bp paired-end were sequenced on an Illumina HiSeq4000 platform. After that the data was demultiplexed to obtain the raw reads, which were evaluated for quality using FastQC, and then filtered using in-house made Perl script before alignment to the poplar reference genomes (Phytozome, http://phytozome.jgi.doe.gov/pz/portal.html). As *Populus trichocarpa* genome has been well sequenced and annotated, it was used as reference genomes. To obtain better results in our study, firstly, the indexed adapter sequences were removed. Secondly, low quality reads with one end of a paired-end read >5% ‘N’ bases or Phred quality score Q ≤ 30 were filtered.

### Variations of SNPs and indels between the chromosome of CHP and L2025

The obtained data were aligned with *Populus trichocarpa* genome according to Burrows-Wheeler Aligner program^55^, and the duplicate reads were removed by the Picard tools. GATK tools was used to realign and calibrate the binary alignment map files to obtain indels, and variant quality score recalibration was used to obtain high-quality SNPs with QD < 2.0, Read Pos Rank Sum < −20.0, and FS > 200.0. After that, the variants with depth coverage < 10 were removed to acquire the final indels and SNPs, and the distribution of indels and SNPs on each chromosome can be shown with Circos^56^.

### Phylogenetic analysis of MYB family genes between Arabidopsis and poplar

To better screen the candidate R2R3-MYB family genes associated with pigments formation, the R2R3-MYB family genes in Arabidopsis genome and *Populus trichocarpa* genome were aligned to construct the phylogenetic tree MEGA 6. 1000 bootstrap replicates in our results were used with neighbor-joining method.

### The relative expression levels of genes involved in pigments formation

Total RNA was extracted from finely ground leaves by the method described by Li^57^, the concentration of which was measured by BioPhotometer (Eppendorf). To explore the relative expression levels of genes involved in pigments formation, qRT-PCR was performed on Applied Biosystems 7500. The synthesis of cDNA was conducted with reverse transcription system (Promega). Gene-specific primers were designed based on the sequence of the target genes (Table S8), and *ACTIN2* gene was selected as housekeeping gene^23^. The relative expression level of genes was evaluated by 2^−ΔΔCt^ method^58^, and analyzed by SPSS 17.0 with three biological replicates.

## Conclusions

Compared with leaves of L2025, leaves of CHP had a higher content of anthocyanins and a lower content of total Chl and carotenoids, and the ratio of anthocyanins to total Chl in CHP was more than 10 times of those in L2025, which might be the physiological reason why CHP had red leaf color. Three structural genes (flavonol synthase 1 family protein, UDP-glucose flavonoid 3-O-glucosyltransferase 3′ and flavonoid 3-O-galactosyl transferase family protein) and six transcription factors (MYB-related protein Myb4, ttranscription factor GAMYB, PtrMYB179, transcription factor bHLH53, transcription factor bHLH3, VARICOSE family protein) might be involved in the anthocyanin synthesis pathway. The above results could provide reference to explore the molecular regulation mechanism of leaf coloration in *Populus deltoids*, and also provided insight into coloration of other woody plants.

## Supplementary information

Dataset 1
