# Supplementary material for: Integrated physiological and genomic analysis reveals structural variations and expression patterns of candidate genes for colored- and green-leaf poplar
Source: Sci Rep. 2019 Aug 1;9:11150. doi: 10.1038/s41598-019-47681-9 (PMC6673700; doi:10.1038/s41598-019-47681-9)
Supplement: Supplementary file 1 — Dataset 1 [file 41598_2019_47681_MOESM1_ESM.docx]

# Integrated physiological and genomic analysis reveals structural variations and expression patterns of candidate genes for colored- and green-leaf poplar

**Weibing Zhuang^1 *^, Hongxue Wang^2^, Tianyu Liu^2^, Tao Wang^1^, Fengjiao Zhang^1^, Xiaochun Shu^1^, Henghua Zhai^1^, Zhong Wang^1*^**

**^1^** Institute of Botany, Jiangsu Province and Chinese Academy of Sciences (Nanjing Botanical Garden Mem. Sun Yat-Sen), Nanjing, 210014, China

**^2^** College of Horticulture, Nanjing Agricultural University, Nanjing 210095, China

*Corresponding author

Telephone numbers: +86-25-84397131

Fax numbers: +86-25-84395266

E-mail address: weibingzhuangnj@sina.com

**Table S1.** The types and ratio of nucleotide substitutions in detected homozygote polymorphic SNPs between 2025 and CHP

|  | SNPs | Ratio (%) |
| --- | --- | --- |
| Transitions |  |  |
| A/G | 11906 | 33.0 |
| C/T | 11803 | 32.7 |
| Transversions |  |  |
| A/C | 3208 | 8.9 |
| A/T | 4208 | 11.7 |
| C/G | 1733 | 4.8 |
| G/T | 3213 | 8.9 |
| Total | 36071 | 100 |

**Table S2.** Summary of SNPs between 2025 and CHP on individual chromosomes compared with the reference genome

| Chr. no. | No. of polymorphic SNPs | No. of intergenic regions | Genic region | | | | | No. of  genes |  |
| --- | --- | --- | --- | --- | --- | --- | --- | --- | --- |
|  |  |  | Total | UTR | CDS | | No. of introns |  |  |
|  |  |  |  |  | Synonymous | Non-  synonymous |  |  |  |
| 1 | 94154 | 66421 | 27733 | 3706 | 3549 | 4021 | 16457 | 3631 |  |
| 2 | 49154 | 31311 | 17843 | 2976 | 2346 | 2515 | 10006 | 2396 |  |
| 3 | 56211 | 36010 | 20201 | 2747 | 2648 | 3089 | 11717 | 2049 |  |
| 4 | 54345 | 36392 | 17953 | 2503 | 2605 | 3020 | 9825 | 2132 |  |
| 5 | 56708 | 36258 | 20450 | 3039 | 2774 | 3259 | 11378 | 2332 |  |
| 6 | 64557 | 40721 | 23836 | 3565 | 3050 | 3366 | 13855 | 2603 |  |
| 7 | 36963 | 24731 | 12232 | 1708 | 1701 | 1746 | 7077 | 1364 |  |
| 8 | 50357 | 30141 | 20216 | 3024 | 2638 | 2705 | 11849 | 2085 |  |
| 9 | 33033 | 18968 | 14065 | 2286 | 1929 | 2006 | 7844 | 1563 |  |
| 10 | 55027 | 33173 | 21854 | 3461 | 2778 | 2879 | 12736 | 2361 |  |
| 11 | 42418 | 29297 | 13121 | 1700 | 1891 | 2296 | 7234 | 1511 |  |
| 12 | 35754 | 24348 | 11406 | 1553 | 1598 | 1794 | 6461 | 1328 |  |
| 13 | 33499 | 22482 | 11017 | 1522 | 1374 | 1649 | 6472 | 1256 |  |
| 14 | 39261 | 25545 | 13716 | 1976 | 1818 | 1985 | 7937 | 1653 |  |
| 15 | 39496 | 25986 | 13510 | 1852 | 1809 | 1961 | 7888 | 1345 |  |
| 16 | 32315 | 22283 | 10032 | 1461 | 1449 | 1658 | 5464 | 1201 |  |
| 17 | 38607 | 26039 | 12568 | 1597 | 1967 | 2405 | 6599 | 1428 |  |
| 18 | 38475 | 25068 | 13407 | 1724 | 1870 | 2090 | 7723 | 1388 |  |
| 19 | 38743 | 28013 | 10730 | 1351 | 1399 | 1790 | 6190 | 1192 |  |
| Scaffolds | 62344 | 52629 | 9715 | 924 | 1532 | 2773 | 4486 | 1571 |  |
| Total | 951421 | 635816 | 315605 | 44675 | 42725 | 49007 | 179198 | 36389 |  |

**Table S3.** Summary of indels between 2025 and CHP on individual chromosomes compared with the reference genome

| Chr. no. | No. of polymorphic indels | No. of intergenic  regions | Genic region | | | | No. of genes |  |
| --- | --- | --- | --- | --- | --- | --- | --- | --- |
|  |  |  | Total | UTR | CDS | No. of introns |  |  |
|  |  |  |  |  |  |  |  |  |
| 1 | 21607 | 15711 | 5896 | 1156 | 420 | 4320 | 3183 |  |
| 2 | 12413 | 8740 | 3673 | 870 | 253 | 2550 | 2142 |  |
| 3 | 13499 | 9403 | 4096 | 823 | 315 | 2958 | 1881 |  |
| 4 | 12878 | 9321 | 3557 | 748 | 292 | 2517 | 1901 |  |
| 5 | 13488 | 9451 | 4037 | 914 | 344 | 2779 | 2074 |  |
| 6 | 16311 | 11208 | 5103 | 1144 | 353 | 3606 | 2350 |  |
| 7 | 9202 | 6678 | 2524 | 540 | 173 | 1811 | 1242 |  |
| 8 | 12099 | 7909 | 4190 | 886 | 287 | 3017 | 1881 |  |
| 9 | 8676 | 5814 | 2862 | 659 | 208 | 1995 | 1420 |  |
| 10 | 13660 | 8996 | 4664 | 1040 | 295 | 3329 | 2138 |  |
| 11 | 9365 | 6817 | 2548 | 551 | 194 | 1803 | 1360 |  |
| 12 | 8496 | 6134 | 2362 | 484 | 182 | 1696 | 1200 |  |
| 13 | 7741 | 5459 | 2282 | 456 | 163 | 1663 | 1144 |  |
| 14 | 9079 | 6224 | 2855 | 667 | 195 | 1993 | 1452 |  |
| 15 | 9531 | 6724 | 2807 | 539 | 227 | 2041 | 1229 |  |
| 16 | 7580 | 5527 | 2053 | 441 | 130 | 1482 | 1054 |  |
| 17 | 8248 | 5964 | 2284 | 435 | 162 | 1687 | 1253 |  |
| 18 | 9096 | 6454 | 2642 | 562 | 207 | 1873 | 1279 |  |
| 19 | 8233 | 6183 | 2050 | 400 | 139 | 1511 | 1063 |  |
| Scaffolds | 10705 | 9358 | 1347 | 196 | 186 | 965 | 1277 |  |
| Total | 221907 | 158075 | 63832 | 13511 | 4725 | 45596 | 32523 |  |

**Table S4.** The statistics of UDP-glucosyl transferase family genes associated with anthocyanin biosynthesis carrying polymorphic SNPs between CHP and L2025

| **Gene ID** | **SNP numbers in coding regions** | **Description** |
| --- | --- | --- |
| Potri.019G092700 | 0 | anthocyanidin 3-O-glucoside 2’’-O-glucosyltransferase [*Ipomoea nil*] |
| Potri.016G016800 | 0 | UDP-glucose flavonoid 3-O-glucosyltransferase 6 [*Fragaria* *ananassa*] |
| Potri.006G009900 | 1 | UDP-glucose flavonoid 3-O-glucosyltransferase 3 [*Fragaria* *ananassa*] |
| Potri.016G016600 | 1 | UDP-glucose flavonoid 3-O-glucosyltransferase 6 [*Fragaria* *ananassa*] |
| Potri.014G026300 | 1 | UDP-glucose flavonoid 3-O-glucosyltransferase 6 [*Fragaria* *ananassa*] |
| Potri.016G017000 | 1 | anthocyanidin 3-O-glucosyltransferase 6-like [*Populus* *euphratica*] |
| Potri.016G016200 | 1 | anthocyanidin 3-O-glucosyltransferase 6 [*Manihot* *esculenta*] |
| Potri.011G097900 | 1 | glycosyltransferase family protein [*Populus* *trichocarpa*] |
| Potri.016G017100 | 1 | anthocyanidin 3-O-glucosyltransferase 1 [*Manihot* *esculenta*] |
| Potri.016G016400 | 1 | UDP-glucose flavonoid 3-O-glucosyltransferase 6 [*Fragaria* *ananassa*] |
| Potri.017G041900 | 1 | Anthocyanidin 3-O-glucoside 2’’-O-glucosyltransferase [*Ipomoea* *purpurea*] |
| Potri.006G010000 | 1 | anthocyanidin 3-O-glucosyltransferase 2-like [*Populus* *euphratica*] |
| Potri.008G215000 | 1 | anthocyanidin 3-O-glucoside 2’’-O-glucosyltransferase [*Ipomoea* *purpurea*] |
| Potri.006G007400 | 1 | UDP-glucose flavonoid 3-O-glucosyltransferase 6 [*Fragaria* *ananassa*] |
| Potri.016G014100 | 1 | UDP-glucose flavonoid 3-O-glucosyltransferase 3 [*Fragaria* *ananassa*] |
| Potri.016G016500 | 1 | anthocyanidin 3-O-glucosyltransferase 2-like [*Populus* *euphratica*] |
| Potri.006G007000 | 1 | UDP-glucose flavonoid 3-O-glucosyltransferase 3 [*Fragaria* *ananassa*] |
| Potri.006G007600 | 2 | UDP-glucose flavonoid 3-O-glucosyltransferase 3 [*Fragaria* *ananassa*] |
| Potri.013G143900 | 2 | anthocyanidin 3-O-glucosyltransferase 7-like [*Populus* *euphratica*] |
| Potri.007G030400 | 2 | Glycosyltransferase 1 [*Populus* *tomentosa*] |
| Potri.016G016100 | 2 | UDP-glucose flavonoid 3-O-glucosyltransferase 6 [*Fragaria* *ananassa*] |
| Potri.007G030300 | 2 | glycosyltransferase 2 [*Populus* *tomentosa*] |
| Potri.014G026400 | 3 | UDP-glucose flavonoid 3-O-glucosyltransferase 3 [*Fragaria* *ananassa*] |
| Potri.T178700 | 3 | UDP-glucose flavonoid 3-O-glucosyltransferase 6 [*Fragaria* *ananassa*] |
| Potri.006G007300 | 3 | anthocyanidin 3-O-glucosyltransferase 6-like [*Populus* *euphratica*] |
| Potri.007G029800 | 3 | glycosyltransferase 2 [*Populus* *tomentosa*] |
| Potri.007G030500 | 3 | UTP-glucose glucosyltransferase family protein [*Populus* *trichocarpa*] |
| Potri.016G014400 | 3 | UDP-glucose flavonoid 3-O-glucosyltransferase 3 [*Fragaria* *ananassa*] |
| Potri.016G016300 | 3 | UDP-glucose flavonoid 3-O-glucosyltransferase 6 [*Fragaria* *ananassa*] |
| Potri.017G089000 | 4 | anthocyanidin 3-O-glucosyltransferase 2-like [*Populus* *euphratica*] |
| Potri.002G123700 | 4 | UDP-glucose flavonoid 3-O-glucosyltransferase 7 [*Fragaria* *ananassa*] |
| Potri.016G017300 | 5 | UDP-glucose flavonoid 3-O-glucosyltransferase 3 [*Fragaria* *ananassa*] |
| Potri.014G041900 | 6 | hydroquinone glucosyltransferase-like [*Populus* *euphratica*] |
| Potri.T010800 | 6 | PREDICTED: UDP-glycosyltransferase 88A1-like [*Populus* *euphratica*] |
| Potri.015G027800 | 7 | anthocyanidin 5,3-O-glucosyltransferase [*Rosa* *hybrid*] |
| Potri.015G027700 | 8 | anthocyanidin 5,3-O-glucosyltransferase [*Rosa* *hybrid*] |
| Potri.009G133300 | 15 | flavonoid 3-O-galactosyl transferase family protein [*Populus* *trichocarpa*] |

**Table S5.** The statistics of MYB family genes associated with anthocyanin biosynthesis carrying polymorphic SNPs between CHP and L2025

| **Gene ID** | **SNP numbers in coding regions** | **Description** |
| --- | --- | --- |
| Potri.005G118500 | 5 | transcription factor MYB98 GN=MYB98 [*Arabidopsis thaliana*] |
| Potri.008G166700 | 5 | MYB-related protein Myb4 [*Oryza sativa*] |
| Potri.010G004300 | 5 | transcription factor MYB3-like [*Populus* *euphratica*] |
| Potri.013G130900 | 5 | transcription factor GAMYB [*Oryza sativa*] |
| Potri.017G126000 | 5 | PtrMYB179 [*Populus* *tremula* x *Populus* *tremuloides*] |
| Potri.018G038000 | 5 | MYB-related protein 3R-1 [*Arabidopsis thaliana*] |
| Potri.002G180800 | 6 | protein LHY [*Arabidopsis thaliana*] |
| Potri.012G038300 | 6 | EARLY-PHYTOCHROME-RESPONSIVE1 family protein [*Populus* *trichocarpa*] |
| Potri.012G082000 | 6 | transcription factor MYB98 [*Arabidopsis thaliana*] |
| Potri.006G241700 | 13 | MYB family transcription factor family protein [*Populus* *trichocarpa*] |

**Table S6.** The statistics of bHLH family genes associated with anthocyanin biosynthesis carrying polymorphic SNPs between CHP and L2025

| **Gene ID** | **SNP numbers in coding regions** | **Description** |
| --- | --- | --- |
| Potri.002G032400 | 5 | transcription factor bHLH110 [*Arabidopsis thaliana*] |
| Potri.011G157700 | 5 | transcription factor bHLH53 [*Arabidopsis thaliana*] |
| Potri.015G048000 | 5 | transcription factor BIM1 [*Arabidopsis thaliana*] |
| Potri.015G134300 | 5 | transcription factor bHLH126 [*Arabidopsis thaliana*] |
| Potri.016G037300 | 7 | transcription factor ORG2 [*Arabidopsis thaliana*] |
| Potri.002G042000 | 9 | transcription factor bHLH3 [*Arabidopsis thaliana*] |

**Table S7.** The statistics of WD40 family genes associated with anthocyanin biosynthesis carrying polymorphic SNPs between CHP and L2025

| **Gene ID** | **SNP numbers in coding regions** | **Description** |
| --- | --- | --- |
| Potri.011G122500 | 5 | PREDICTED: WD repeat-containing protein 44-like [Populus euphratica] |
| Potri.010G197700 | 9 | transducin family protein [Populus trichocarpa] |
| Potri.011G166400 | 10 | VARICOSE family protein [Populus trichocarpa] |
| Potri.011G021700 | 28 | hypothetical protein POPTR_0011s00900g [Populus trichocarpa] |

**Table S8.** Specific primers used in relative quantitative real-time RT-PCR

| **Gene name** | **Accession** | **Forward primer (5’to 3’)** | **Reverse primer (5’ to 3’)** |
| --- | --- | --- | --- |
| PtrPAL1 | Potri.006G126800.1 | TTGACTTGAGGCATTTGGAG | CAATGGATAGGTAGCACTGC |
| PtrC4H1 | Potri.013G157900.1 | ACTCTGGGACGTYTGGTACA | GCTTCATAGATTTACAGTGA |
| Ptr4CL1 | Potri.001G036900.1 | TAGTGAAATCAGAAAAGTCT | CGCAAGTATTAAAGAAATAA |
| PtrCHS1 | Potri.014G145100.2 | TAAGGACTTGGCTGAGAACA | ATCAGAGTCAGGAAGGATGG |
| PtrF3'5'H | Potri.007G083700.1 | GCACATTCAGTTCAAGGACG | TGCCTATTTTCTCTTCCAGC |
| PtrCHI1 | Potri.010G213000.1 | GTCACTTKCTGCTAAATGGA | GCCAATCATTGACTCTAGCA |
| PtrF3H1 | Potri.005G113900.1 | CGCACCAGAGACTATTCAAG | TCCAAGTGTAAGGTCTGGTT |
| PtrDFR1 | Potri.002G033600.1 | CCTGACAGCACTTTCATTGA | ACACGCCAAATTCTCATCAA |
| PtrANS1 | Potri.003G119100.1 | GGTGACACTRTTGAGATCTT | CCATTTCAACGACATASCTT |
| PtrF3'H | Potri.003G066400.1 | TTGCCACTGTATCCCTTATC | AGAGAAGATAACATCGTGGG |
| PtrLAR1 | Potri.008G116500.1 | CAATCAATGGCCYTGATGAT | TGTCGTCCAAGAAAAAGAGA |
| PtrANR1 | Potri.004G030700.1 | TTTGCTTCTGAGGATCCTGA | TCTCAGCTAGTGTCTTGGAG |
| PtrUFGT1 | Potri.013G118700.1 | CAACAACTCCATCTTCTCCA | ACCAAACAACTCACCTTTCT |
| PtrFLS1 | Potri.004G139500.1 | TGAGGAGTTCATTATGCCAG | CATCCGCAATCAAACCCACT |
| Shikimate O-hydroxycinnamoyltransferase-like | Potri.005G028000.1 | CGAAGACACGCCAGAAAAAAG | TCAGAGCCTCCTTGAGCACTT |
| UDP-glucose flavonoid 3-O-glucosyltransferase 3 | Potri.016G017300.1 | TAGTCTTCATACCGACACCAG | CTGATACGATTACTGGCTGTC |
| Hydroquinone glucosyltransferase-like | Potri.014G041900.1 | TCATCACCACCAGCAACGAAG | TGATTTCAGGGACTTGAGGCT |
| Predicated: UDP-glycosyltransferase 88A1-like | Potri.T010800 | CATAACCAAACTACCAAGAGC | AAGTGTTCAGTATCATCCCCT |
| Anthocyanidin 5,3-O-glucosyltransferase | Potri.015G027800.1 | TACAATCATAGTCTCCGCCAT | AAGAGTTATGGAGGGATTGG |
| Anthocyanidin 5,3-O-glucosyltransferase | Potri.015G027700.1 | ATGACCCCTACTTTTCCACAC | TAAGAGGGTCTGGTGGAGGTT |
| Flavonoid 3-O-galactosyl transferase family protein | Potri.009G133300.1 | ATGGTTGGATAGTCAAAAGGC | GCTGATAAACGCCACAGACTT |
| Transcription factor MYB98 | Potri.005G118500.1 | TTTGGTGTCCTTACTGGCTCT | ATGAGGTTTTCTTGAGGGAGG |
| MYB-related protein Myb4 | Potri.008G166700.1 | CAAGATTACCAGGAAGGACAG | GACTTGGGTTCGCCATTTTGT |
| Transcription factor MYB3-like | Potri.010G004300.1 | GAAAGGTGCGGAAAGAGTTGC | ACTGACTGAAGTTGGATTACG |
| Transcription factor GAMYB | Potri.013G130900.1 | GGAACACAAGAACAAAGAGAC | TTGGGTGGTCGGGAAAGTAAA |
| MYB-related protein 3R-1 | Potri.018G038000.1 | CAAGGGACAGGTGATAGTG | TTGCTCTGATAAAGTGCCC |
| Protein LHY | Potri.002G180800.1 | GAAGGAGGCTGTTGCTAAAG | CTTTGCTCCTGCCTGTGATG |
| Early-phytochrome-responsive 1 family protein | Potri.012G038300.1 | TATTTCTCTAAGGTGGTCCG | AGCCAAAGCAGATAAAACGG |
| Transcription factor MYB98 | Potri.012G082000.1 | GTTTCCAGAGTGGTGGCTAC | GTCTTTCTTGCGAAGGGTTG |
| MYB family transcription factor family protein | Potri.006G241700.1 | GGATGTTATGTTTGACGAGA | CATCACTATCACCTACCCCT |
| PtrMYB179 | Potri.017G126000.1 | TGAGTTCATCAGGAATAAGG | TAATCAAGTCTTCTTCGTCC |
| Transcription factor bHLH110 | Potri.002G032400.1 | ACAGAAATACGAGAGTCCAG | GGAAAAGAATCCGATGAGAG |
| Transcription factor bHLH53 | Potri.011G157700.1 | TCCTACCACTACCCGAAACG | TCTGGGAGTCCTGGATTGGG |
| Transcription factor BIM1 | Potri.015G048000.1 | AGTCAGCCAAAGGTAGTAAC | TTATGGATAGGAGAAGGGGT |
| Transcription factor bHLH126 | Potri.015G134300.1 | GATTTCCTCCAGTCCACACC | GCCGCCGACCTTTCTCCATA |
| Transcription factor ORG2 | Potri.016G037300.1 | GTATTCTTCACTCCGTTCAC | TCAGTCTCTCCACTTGTTGT |
| Transcription factor bHLH3 | Potri.002G042000.1 | TTCTGGTATGCTTCTGGCTT | CTTTTCTTTTCTCTACTCCC |
| Predicated: WD repeat-containing protein 44-like | Potri.011G122500.1 | GAGGGATTGGAGGTGGGGTT | TATGCCTTGTCACACTATTC |
| Transducin family protein | Potri.010G197700.1 | CGGATAGCAGGGACAGCATT | GGTATGACCCCTCAACGATT |
| VARICOSE family protein | Potri.011G166400.1 | AACTCATCTAATAACCCCT | AGATTTTTTCCTTGTTTTC |
| Hypothetical protein POPTR_0011s00900g | Potri.011G021700.1 | AAGAATACAGTTATCCAGGG | ACGGACCCATTTCTTTTTAG |
| PtrACTIN2 | Potri.019G010400.1 | GCCATCTCTCATCGGAATGGAA | AGGGCAGTGATTTCCTTGCTCA |


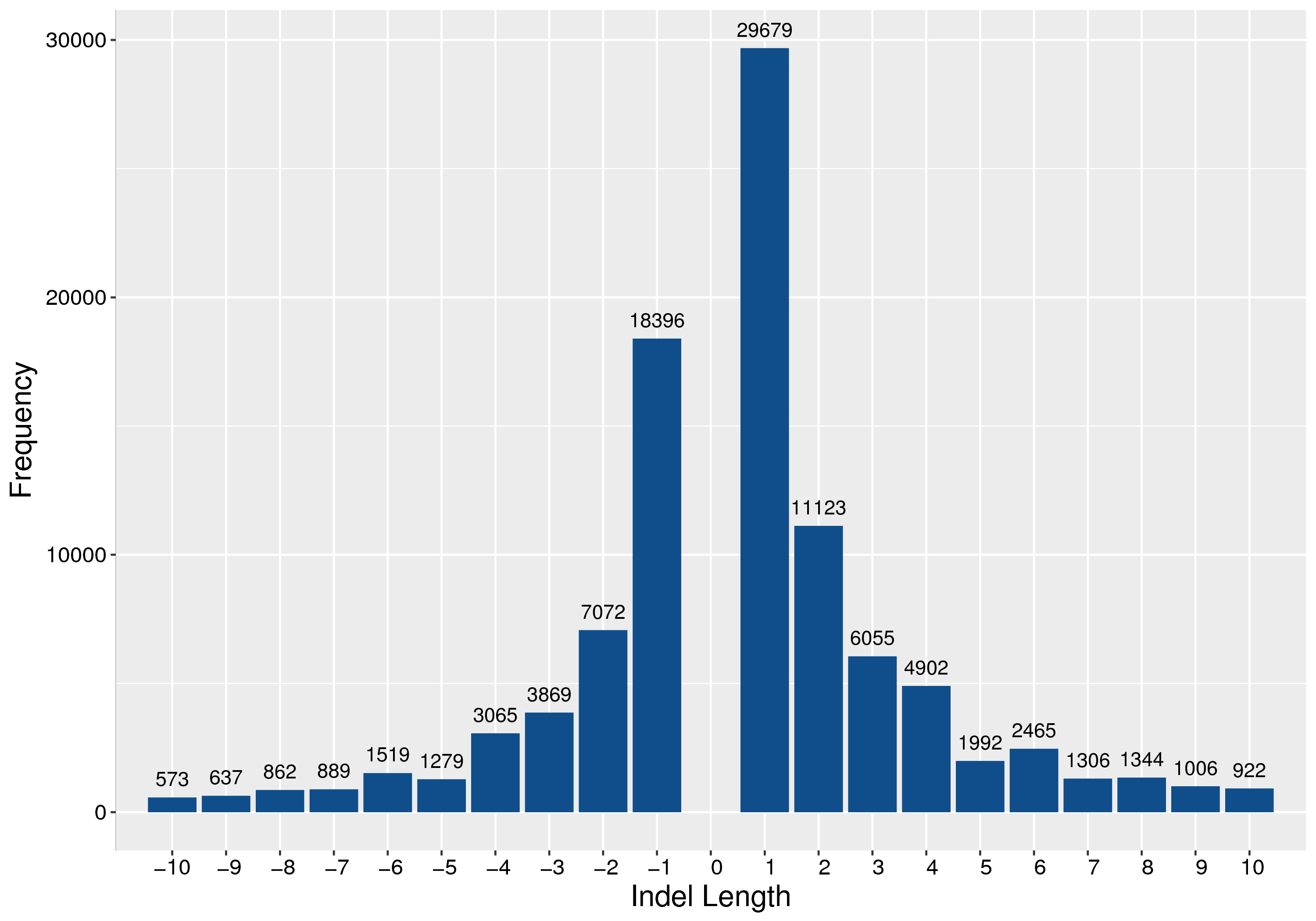


**Figure S1.** The lengths and corresponding frequency of indels between 2025 and CHP genome sequence


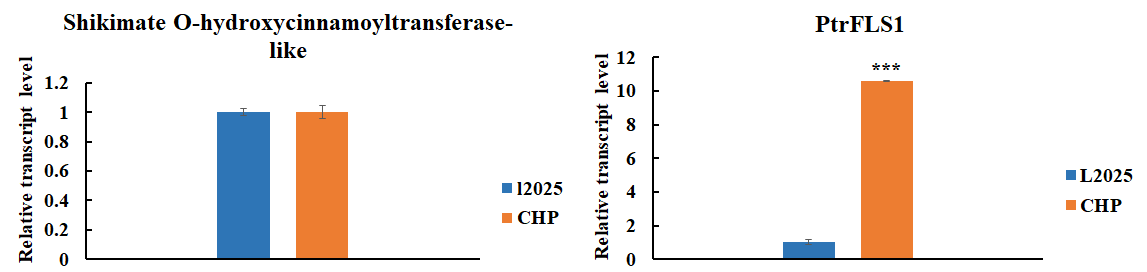


**Figure S2.** The relative expression levels of two genes involved in flavonoid biosynthesis in the leaves determined by quantitative real-time PCR (qRT-PCR) analysis between L2025 and CHP. Bars indicated the mean ± SE, n= 3. *** indicated P ⩽ 0.001


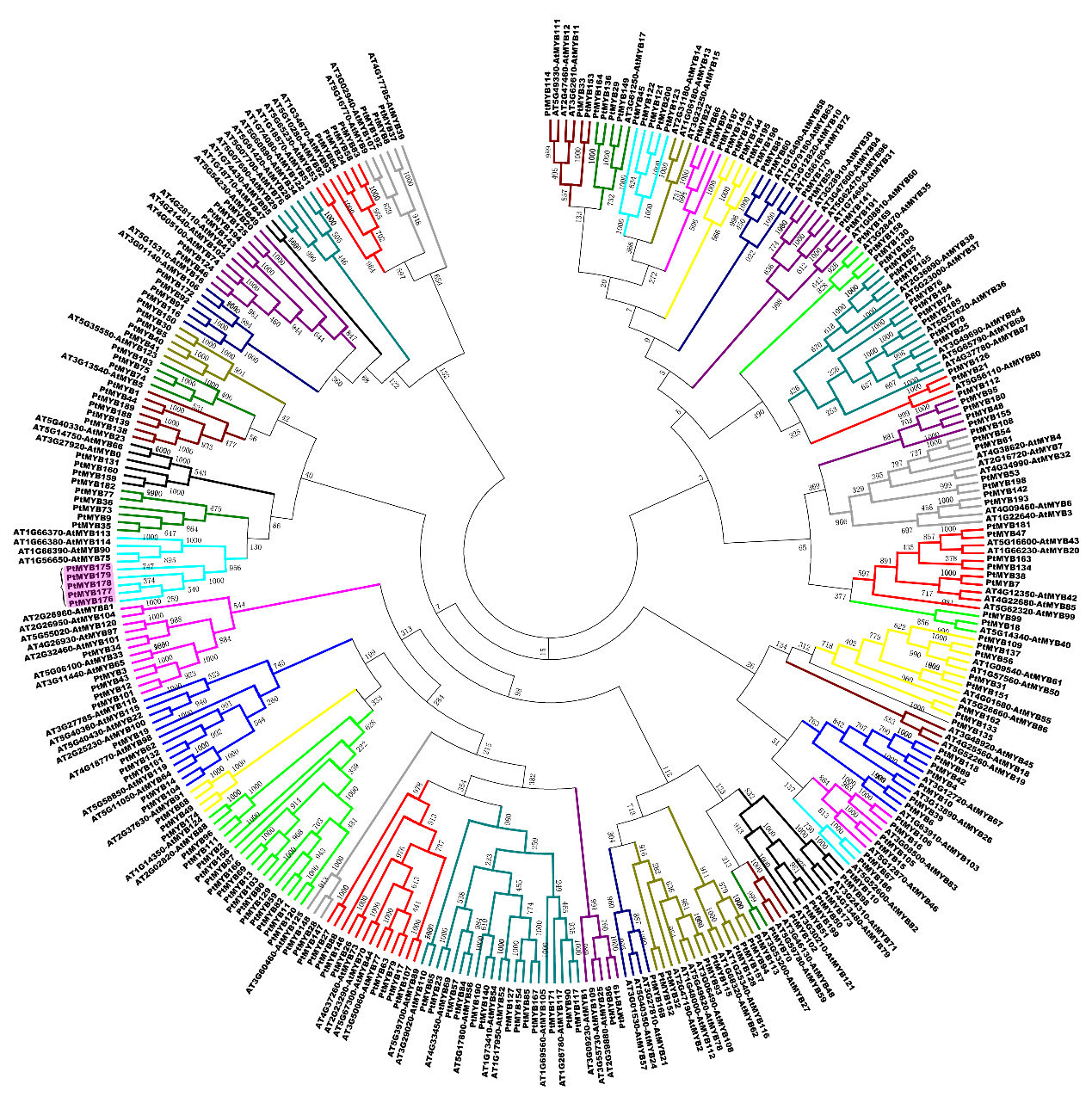


**Figure S3.** Phylogenetic analysis of R2R3-MYB transcription factors between *Populus trichocarpa* and *Arabidopsis thaliana*. *PtrMYB179* was grouped into *PAP1* and *PAP2* of *Arabidopsis thaliana*, which were marked in purple color. The phylogenetic tree was constructed using MEGA 6 with 1000 bootstrap replicates. Numbers indicate the percentage of consensus support
